# The use of echocardiographic and clinical data recorded on admission to simplify decision making for elective percutaneous coronary intervention: a prospective cohort study

**DOI:** 10.1186/s12911-019-0797-9

**Published:** 2019-03-18

**Authors:** Rabah M. Al abdi, Hussam Alshraideh, Heba H. Hijazi, Mohamad Jarrah, Mohammad S. Alyahya

**Affiliations:** 1 Biomedical Engineering Department, Faculty of Engineering, Jordan University of Science and Technology, Irbid, Jordan; 2 Industrial Engineering Department, Faculty of Engineering, Jordan University of Science and Technology, Irbid, Jordan; 3 Department of Health Management and Policy, Faculty of Medicine, Jordan University of Science and Technology, Irbid, Jordan; 4 Division of Cardiology, Internal Medicine Department, King Abdullah University Hospital, Jordan University of Science and Technology, Irbid, Jordan

**Keywords:** Percutaneous coronary intervention, Quality of life, Risk factors, Elective percutaneous coronary intervention, Decision making

## Abstract

**Background:**

Coronary artery disease (CAD), a leading cause of mortality, affects patient health-related quality of life (HRQoL). Elective percutaneous coronary interventions (ePCIs) are usually performed to improve HRQoL of CAD patients. The aim of this study was to design models using admission data to predict the outcomes of the ePCI treatments on the patients’ HRQoL.

**Methods:**

This prospective cohort study was conducted with CAD patients who underwent ePCIs at the King Abdullah University Hospital in Jordan from January 2014 through May 2015. Six months after their ePCI procedures, the participants completed the improved MacNew (QLMI-2) questionnaire, which was used for evaluating three domains (physical, emotional and social) of HRQoL. Multivariate linear regression was used to design models to predict the three domains of HRQoL from echocardiographic findings and clinical data that are routinely measured on admission.

**Results:**

The study included 239 patients who underwent ePCIs and responded to the QLMI-2 questionnaire. The mean age (± standard deviation) of the participants was 55.74 ± 11.84 years, 54.58 ± 11.37 years for males (*n* = 174) and 59.11 ± 12.49 years for females (*n* = 65). The average scores for physical, emotional and social HRQoL were 4.38 ± 1.27, 4.4 ± 1.11, and 4.37 ± 1.32, respectively. Out of the 42 factors inputted to the models to predict HRQoL scores, 10, 9, and 9 factors were found to be significant determinants for physical, emotional and social domains, respectively, with adjusted coefficients of determination of 0.630, 0.604 and 0.534, respectively. Basophil levels on admission showed a significant positive correlation with the three domains of HRQoL, while aortic root diameter showed a negative correlation. Scores for the three domains were significantly lower in women than in men. Hypertensive and diabetic patients had significantly lower HRQoL scores than patients without hypertension and diabetes.

**Conclusion:**

The prediction of HRQoL scores 6 months after an ePCI is possible based on data acquired on admission. The models developed here can be used as decision-making tools to guide physicians in identifying the efficacy of ePCIs for individual patients, hence decreasing the rate of inappropriate ePCIs and reducing costs and complications.

## Background

The accumulation of atherosclerotic plaque in the coronary arteries narrows the blood vessels and leads to coronary artery disease (CAD), which decreases blood perfusion to the heart muscles. The leading cause of morbidity and mortality worldwide is CAD [1]. In 2010, more than 75% of deaths due to CAD were attributed to low- and middle-income countries [2, 3]. Jordan is considered a middle-income country [4].

The progression of CAD can lead to heart failure, angina pectoris and myocardial infarction (MI). Percutaneous coronary intervention (PCI) is usually performed to treat CAD. While primary PCI is very urgent in patients with acute MI (heart attack), elective PCI (ePCI) is also used for CAD patients to relieve symptoms that may be difficult to control with medication and to improve their health-related quality of life (HRQoL). The number of PCI procedures performed annually worldwide exceeds 3 million [5], and in Jordan, it exceeds 6000 [6]. Despite the survival advantages of PCI over drug therapy [7], multiple studies have shown that there was no mortality advantage of PCI to stable CAD [8].

The HRQoL for patients undergoing ePCI procedures in Jordan has not been studied [9]. Predicting the risk of low HRQoL in patients who are undergoing ePCIs is an essential component of an effective PCI [10]. The aim of this study was to 1) evaluate the three domains of HRQoL (physical, emotional and social) for Jordanian patients 6 months after their ePCI procedures and 2) design models based on sociodemographic and clinical information gathered on admission to predict their HRQoL 6 months after ePCI procedures.

## Methods

### Study design and sample

This project was a prospective cohort study conducted with patients who underwent ePCIs (identified from their discharge summary) in the King Abdullah University Hospital (KAUH) over an 18-month period (January 1, 2014 to May 31, 2015). The HRQoL was assessed for these patients 6 months after their ePCI procedures. A six-month period after the ePCI was selected since it is widely used as a community norm of postinfarction [10–12]. KAUH is located in Ar Ramtha, Jordan, and it is considered the largest medical structure in the northern area of Jordan and serves more than 1 million people. In addition, KAUH is a teaching hospital affiliated with the Jordan University of Science and Technology.

The study subjects were patients 22 years of age or older who were admitted as inpatients to the Department of Internal Medicine in KAUH for PCI procedures. Participants recruited into the study were those who met the following criteria: patients who underwent ePCIs without severe cognitive impairments; and patients without serious comorbidities (such as cancer, liver failure, chronic obstructive pulmonary disease and kidney failure), which may have a greater effect on HRQoL than cardiac diseases. Patients admitted for a primary PCI were excluded from the study. No exclusions were made for an upper age limit. No specific intervention was studied in these patients during the follow-up period.

Each patient was called to set up an appointment to have a telephone administration of the 26-item QLMI-2 and nine sociodemographic questions. The patients’ phone numbers were retrieved from the electronic medical records in the hospital. Patients who agreed to participate were told that this is a voluntary study and that the patients can refuse to participate or withdraw from the study at any time without question. Upon the patients’ approval, a research assistant administered the questionnaire over the telephone.

The research assistant who conducted the study was a female junior house officer. She received extensive training on the QLMI-2 questionnaire. At the beginning of each call, the research assistant told the patient that participation in this study (questionnaire) was voluntary and that he or she was free to withdraw from answering the questionnaire at any time. Answers to the questions in the questionnaire were collected via a telephone interview. The calls were conducted in a quiet private room where nobody from the outside could hear the dialogue. In addition, the research assistant ensured the participants’ privacy by recording all answers using anonymous identification numbers without participant names. The average time for administering the questionnaire was 14 min per patient.

Ethical approval for the study was obtained from the Institutional Review Board (IRB) committees at the Jordan University of Science and Technology and the KUAH. All patients’ personal data, such as the names and addresses extracted from the medical records, were treated confidentially, and all other study data were indexed using anonymous study ID numbers.

### Review of medical records

Data on the clinical and sociodemographic factors that could affect the HRQoL after the ePCI were collected from the medical records of the participating patients. Medical records available in the database of the hospital were reviewed to find clinical, echocardiographic, electrocardiographic, demographic, and laboratory information. Data were collected on admission, and PCI features were collected from the discharge summary reports after the procedure.

The clinical factors included the number of comorbidities, number of medications, body temperature, mean heart rate and noninvasive blood pressure (systolic and diastolic) at admission and length of stay (LOS) in the hospital following the procedure. The echocardiographic factors included the left ventricle ejection fraction (LVEF), septal thickness, aortic root diameter, left ventricle end-systolic diameter (LVES), left ventricle end-diastolic diameter (LVED), posterior left ventricle wall thickness, and left atrial diameter. The laboratory factors included cardiac enzymes, lipid profiles, chloride concentration, urea concentration and basophil percentage. The electrocardiograph (ECG) readings included QTc, P, R and T results. Several of the above factors were documented to determine their correlation with the HRQoL of CAD patients [13–15].

### Questionnaire

After gaining approval from the original author, the improved MacNew Quality of Life following Acute Myocardial Infarction (QLMI-2) questionnaire was used to measure the HRQoL of the patients 6 months after their ePCI procedures [13]. The QLMI-2 questionnaire is a self-administered HRQoL instrument related to three domains of HRQoL: physical, emotional and social. This questionnaire was originally designed to evaluate the effect of CAD and its treatments on the patients’ QoL [16]. In this study, this questionnaire was used to assess the correlation of ePCI procedures with everyday activities related to the HRQoL. The QLMI-2 has the well-established psychometric property of sensitivity to change [17]. The internal consistency, validity and reliability of QLMI-2 have been demonstrated in patients with CAD [18]. The QLMI-2 consists of 27 items to measure the three domains of HRQoL. A Likert scale from 1 to 7 was used to rate each item with higher scores indicating better health status.

Three health psychologists translated the English version of QLMI-2 into the Arabic language to lessen the barriers of assessment with patients. A pilot test of the translated QLMI-2 was conducted on 28 participants within the target population to ensure that the tool can be understood by those who will use it, and most patients easily understood and answered questions in the Arabic version of QLMI-2. In addition, most patients received the same score in the first and second trials of answering the questionnaire. However, more than three-fourths of the patients did not answer the question about sexual intercourse (question 27). This question is inappropriate in the Jordanian setting. Hence, question 27 was omitted. Two experts in nursing reviewed the final version of the questionnaire to assess its content validity, comprehensiveness, relevance and clarity, and they recommended no modifications.

In addition to the 26 QLMI-2 items, 9 sociodemographic questions were asked that concerned age, smoking history, residency (city or town), marital status, level of education, employment status, average monthly family income (in Jordanian dinar), number of family members and the patient’s type of medical insurance (government, military or other). The questionnaire with 35 questions, 26 from QLMI-2 and 9 sociodemographic questions, was administered to each subject 6 months after his/her ePCI procedure [13].

### Statistical analysis

Numerical factors are presented with their associated minimum, maximum, mean, standard deviation, skewness and kurtosis. Categorical factors are presented as frequencies and percentages. Differences in mean values were assessed using either the t-test or Mann-Whitney U test for normally and nonnormally distributed variables, respectively. A significance level of 95% with a two-sided test was used in the analyses as appropriate to indicate statistical significance. To identify significant predictors of the three domains of HRQoL (physical, emotional and social), multivariate linear regression (MLR) was used in combination with stepwise selection criteria. The factors collected on admission were used as independent variables in the MLR, and *p* < 0.05 was used to include factors in the MLR. Each of the three domains of HRQoL was considered as an outcome variable of interest and thus used as a dependent variable in the MLR.

Before performing MLR analyses, the problem of multicollinearity between the independent variables was reduced by checking pairwise Pearson correlations (*r*) between them. If *r* > 0.7 was found between any pair of independent variables, only one of the two variables was used in the MLR. The variable that was used was the one with more medical or clinical meaning or most frequently measured on admission. Statistical analyses were performed using SPSS version 19 (IBM, Chicago, IL).

### Demographics

**Fig. 1:**
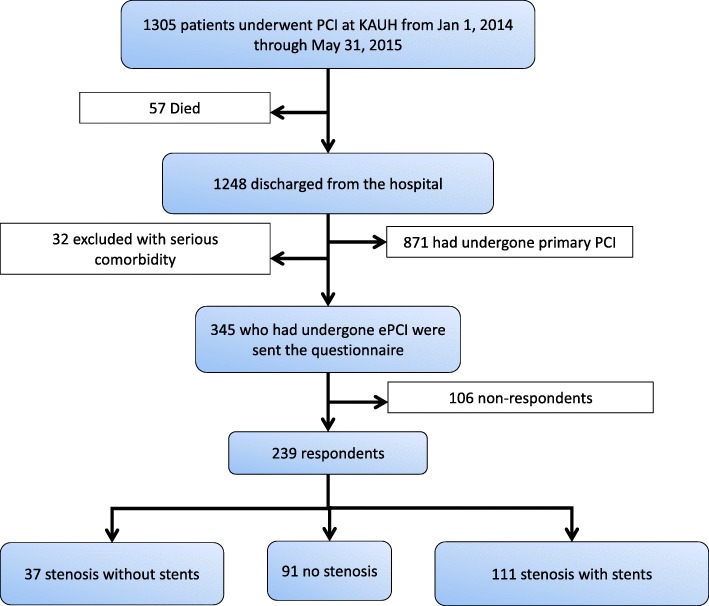
Flow chart of the study population and patient inclusion criteria. The included patients were stratified based on PCI features

Figure 1 shows the flow chart for the selection of the study population. From January 2014 to May 2015, 1305 patients who had been referred by cardiologists for PCIs were recruited for the study. Of the patients recruited, 57 died before the end of the follow-up period (6 months) after their PCI procedures. Patients who underwent primary PCIs (*n* = 871) were excluded from the study. Thirty-two patients were excluded because they had serious comorbidities, including cancer (*n* = 7), kidney failure (*n* = 12) or chronic obstructive pulmonary disease (COPD; *n* = 13).

Of the remaining 345 patients who had been invited to participate and were sent questionnaires, 239 patients agreed to participate in the study and answer all items in the questionnaire, resulting in a response rate of 69.3%. The respondents were stratified as follows based on their ePCI results: stenosis (*n* = 148) and no stenosis (*n* = 91). Patients with stenosis were stratified as follows based on stent deployment: stents deployed (*n* = 111) and no stents deployed (*n* = 37). The patients were categorized based on their LVEFs as normal (LVEF > 0.5), intermediate (0.4 < LVEF < 0.5), and reduced (LVEF < 0.4) LVEFs [19].

## Results

### Baseline characteristics

The mean age of the respondents (55.7 years) was similar to that of the nonrespondents (55.1 years; *p* = 0.52). The respondents had a shorter average length of stay than the nonrespondents (2.90 days vs 3.62 days; *p* < 0.01). The proportion of respondents who had stents deployed (111 of 239, 46.4%) was higher than that of the nonrespondents (34 of 106, 32.1%). A total of 239 patients underwent ePCIs and answered the 35-question survey, as summarized in Tables 1 and 2.

**Table 1:** Summary of patients’ characteristics

Factor	Categories	Frequency	Percent
Gender	Female	65	27.2
	Male	174	72.8
Smoking	Never	98	41.0
	Smoker	84	35.1
	Ex-Smoker	57	23.9
Education	Illiterate	65	27.2
	Primary school	95	39.8
	Secondary school	51	21.3
	University	28	11.7
Employment	Full time	89	37.2
	Housewife	28	11.7
	No work	50	20.9
	Part-time	21	8.8
	Retired	51	21.3
Monthly Income (JD)	< 200	54	22.6
	200 to 400	58	24.3
	400 to 800	101	42.3
	more than 800	26	10.9
Residency	City	144	60.3
	Village	95	39.7
Medical Insurance	Government	134	56.1
	Military	53	22.2
	Other	52	21.8
Marital status	Married	232	97.1
	Single	7	2.9
Chronic diseases	HTN	129	54.0
	DM	80	33.5
	Others	40	16.7

**Table 2:** Numerical summary of patients’ data

Factor	Min	Max	Mean	Std.	Skewness	Kurtosis
AGE	30	81	55.74	11.840	.365	−.701
No. family	0	14	5.29	3.009	.358	−.641
Max LOS	1	15	2.94	1.516	5.168	34.240
No. admission	1	3	1.14	.406	2.958	8.520
No. medications prior to admission	0	11	5.24	2.299	.186	−.239
No. Comorbidities	0	5	1.08	.958	.743	.566
Body temperature	36.0	38.5	36.862	.399	.505	3.037
Heart rate	44.0	145.0	71.653	13.242	1.351	4.270
Min PTINR	.80	2.09	.9896	.151	3.811	18.990
Max ALT	3.61	3325.00	59.2251	266.302	10.446	115.732
Max AST	4.8	6466.0	71.622	427.7110	14.278	212.567
Max chloride	130.0	158.0	141.113	3.592	.608	3.025
Max CK	14.0	9035.0	489.310	1080.080	4.663	26.733
Max CK MB	13.9	477.6	47.980	66.608	3.517	14.042
Max PTINR	.80	6.78	1.1268	.545	6.958	59.259
Max TRIGLYCERIDES	.57	13.94	2.5591	1.716	2.247	8.436
Max urea	2.70	48.00	8.4600	6.040	3.030	12.662
Gamma GT	8.0	532.0	55.238	72.598	4.301	21.700
Basophil (%)	.1	2.7	.650	.439	1.526	2.785
QTc	249.0	507.0	407.204	24.453	−.294	8.528
Axes P	− 88.0	999.0	83.793	151.835	4.696	22.965
Axes R	−87.0	257.5	34.312	41.573	.803	5.897
Axes T	−89.0	174.0	46.592	37.751	.010	2.505
Septal thickness	.7	1.3	1.103	.146	−.640	.276
Aortic root diameter	2.5	4.5	3.301	.337	.543	1.224
LVEF	.24	.61	.5294	.102	−1.317	.439
LVED	4.5	7.3	5.124	.382	2.863	9.556
LVES	3.0	6.1	3.612	.513	2.532	6.114
Posterior LV wall	1.1	1.3	1.144	.068	1.278	.285
Left atrial diameter	3.4	5.9	3.721	.355	2.714	10.184
Max BP diastolic	60.0	110.0	82.611	9.453	.411	.250
Max BP systolic	100	190	126.59	14.518	1.505	2.812
BMI	18.3	72.0	29.499	6.533	2.416	10.911

The mean age of patients included in this study was 55.70 ± 11.84 years with 30 and 81 years as the minimum and maximum ages, respectively. Of these participants, 174 were males (72.8%), and 65 were females (27.2%). There was a significant difference (*p* = 0.01) between the mean age of male participants (54.48 ± 11.37 years) and the mean age of female participants (59.11 ± 12.49 years). Table 1 shows that the majority of the participants were either current or previous smokers (35.1 and 23.8%, respectively), while 41% were never smokers. Approximately 32.6% of the participants were housewives or had no job, and 21.3% were retired. The participants were distributed based on their education levels, with 11.7% who had university degrees and 27.2% who were illiterate. Only 10.9% of the participants had a monthly income of 800 Jordanian Dinar (JD) or more. The average number of persons per family was 5.29 (excluding the parents, as in the father and mother).

As shown in Table 2, the participants had an average length of stay in the hospital of 2.9 days, an average of 1.08 comorbidities, and were prescribed an average of 5.24 medications per patient prior to admission. Fifty-four percent of the participants had hypertension (*n* = 129), and 33.5% had diabetes (*n* = 80). The number of participants who had both hypertension and diabetes was 61, composing 47% of the patients with hypertension and 76% of the patients with diabetes. The distribution of diagnoses based on the participants’ PCI results was 38.1% of the patients were normal (no stenosis found), 15.5% had stenosis without stent deployment and 46.4% had stenosis with stent deployment. Based on body mass index (BMI), only 18% of the patients had normal weight (18.5 < BMI < 25), and the rest were either overweight (44%; 25 < BMI < 30) or obese (37.6%; BMI > 30) [20]. The average LVEF value was 0.5294. The number of participants with normal LVEFs was 163 (68.2%), with intermediate LVEFs was 28 (11.7%) and with reduced LVEFs was 48 (20.1%) [21].

### Health-related quality of life (HRQoL) scores

The HRQoL scores for the 239 patients were calculated following the standard analysis procedure of the MacNew QLMI-2 questionnaire [13]. Scores were calculated for the three HRQoL domains. The scores for all domains ranged from 1 to 7, with averages of 4.38 ± 1.27, 4.4 ± 1.11, and 4.37 ± 1.32 for the emotional, physical and social domains, respectively. Females had significantly lower average scores than males for all HRQoL domains (*p* < 0.05). The mean scores for females were 3.97 ± 1.22, 4.07 ± 1.13 and 4.00 ± 1.36 for the physical, emotional and social domains, respectively, while the mean scores for males were 4.52 ± 1.28, 4.50 ± 1.16 and 4.47 ± 1.40 for the physical, emotional and social domains, respectively.

**Fig. 2:**
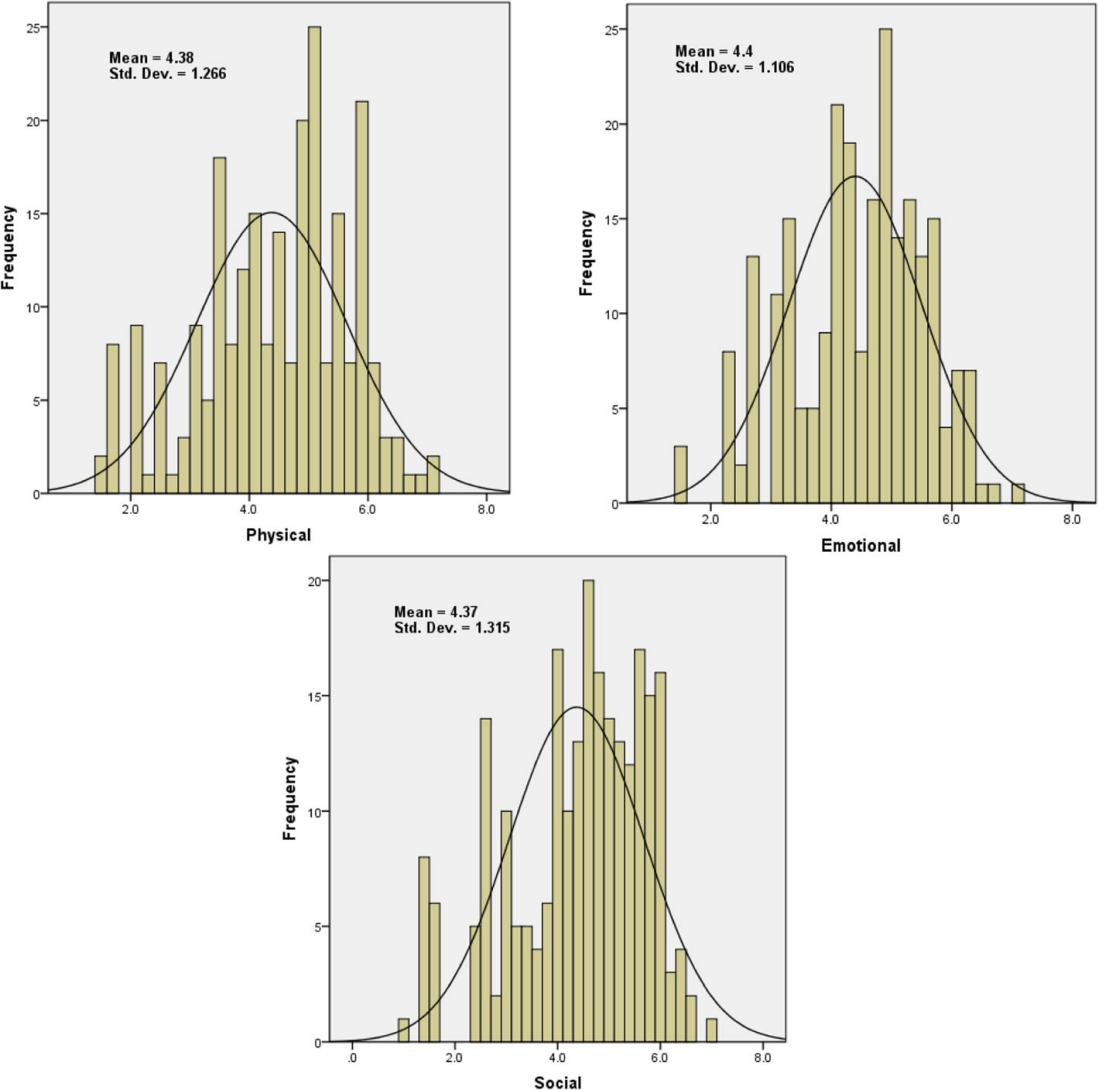
Histogram with normal curve fits of the scores for the three HRQoL domains

Histograms with normal curve fits for HRQoL scores are shown in Fig. 2. The figure shows that the density functions of the physical, emotional and social HRQoL scores are skewed to the left. Surprisingly, approximately 49% of those with a physical HRQoL score less than 4 had no stenosis, and 33% had stenosis with stent deployment. Approximately 45, 38 and 37% of females had scores of less than 4 in the physical, emotional, and social domains, respectively, while 22.6, 26 and 28% of males had scores of less than 4 in the physical, emotional, and social scores, respectively.

**Fig. 3:**
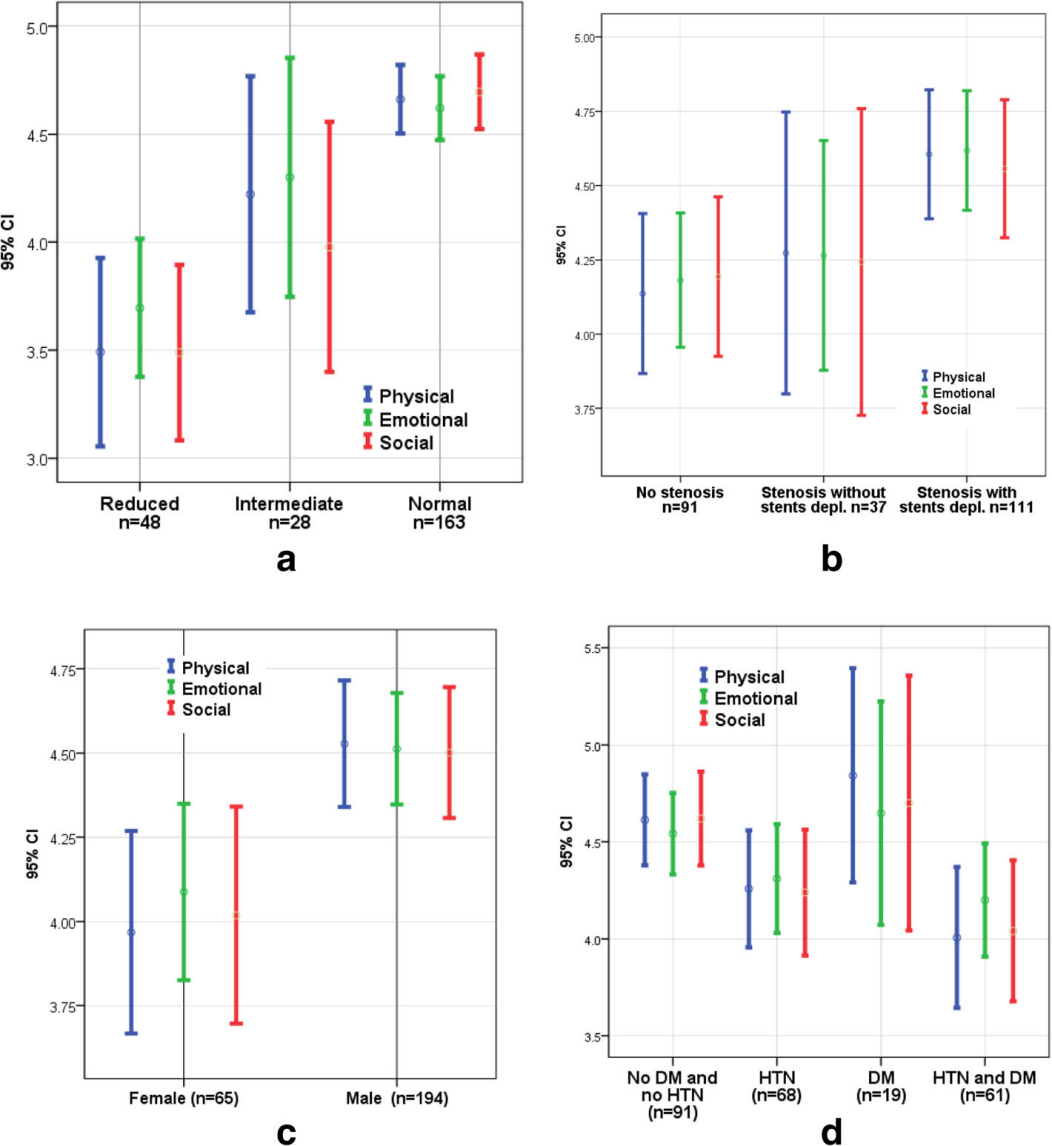
Mean scores with 95% confidence intervals for the three domains of HRQoL for participants grouped according to (**a**) left ventricle ejection fraction, (**b**) ePCI features, (**c**) sex and (**d**) the presence of HTN and DM individually or in combination

Figure 3(a) shows the mean HRQoL scores of the three groups of participants based on their LVEF values (normal, intermediate or reduced) with 95% confidence intervals. The scores of the three domains of HRQoL were significantly lower for participants with reduced LVEF than for participants with normal LVEF (*p* < 0.05). The physical and emotional HRQoL scores for participants with intermediate LVEF were significantly higher (p < 0.05) than those for participants with reduced LVEF, but the social scores were not significantly different. Only the social HRQoL score for participants with intermediate LVEF was significantly lower than the scores for participants with normal LVEF.

Figure 3(b) shows the mean HRQoL scores for the three groups of participants based on their ePCI features with 95% confidence intervals. The groups were stratified as patients with no stenosis, those with stenosis without stent deployment, and those with stenosis with stent deployment. The scores for the three domains of HRQoL for participants with no stenosis were significantly lower than those for participants with stenosis with stent deployment (*p* < 0.05). The differences in the other HRQoL scores between groups were not significant. Figure 3(c) shows the HRQoL scores stratified based on sex. The physical, emotional and social HRQoL scores were significantly lower for women than for men (*p* = 0.002, 0.007 and 0.012, respectively). Figure 3(d) shows the HRQoL scores for the following four groups of participants based on the presence of hypertension (HTN) and diabetes (DM): HTN and DM free, HTN alone, DM alone and HTN with DM. The physical and social HRQoL scores were significantly higher for participants without HTN and DM than for participants with both HTN and DM.

### Predictors of the HRQoL scores

To identify significant predictors of HRQoL scores, MLR was used in combination with stepwise selection criteria. The significant predictors identified for each score type are shown in Table 3. The total number of predictors used in the MLR to predict the HRQoL scores was 42 (33 scalar and 9 categorical). Of the scalar predictors, 11 were taken from laboratory results, seven from echocardiography measurements and four from ECG parameters. Excluding the constant in the linear regression, the present study identified 10 significant predictors (*p* < 0.05) for the physical score, 9 significant predictors for the emotional score and 9 significant predictors for the social score.

**Table 3:** Estimated MLR coefficients of scores for the three domains of HRQoL

Data Category	Parameter	Physical		Emotional		Social	
		B	sig	B	sig	B	sig
	(Constant)	6.644	.000	17.658	.000	10.317	.000
Echocardiology	Aortic root diameter	−.648	.002	−1.054	.000	−.703	.002
	Septal thickness	−1.273	.005	−1.258	.001		
	LVES	.655	.000				
	LVEF	3.142	.000			1.622	.012
Clinical	Max BP diastolic	−.026	.000				
	Max BP systolic			−.018	.000	−.021	.000
	BMI			−.023	.006		
	AGE	−.013	.022			−.027	.000
Laboratory	Basophils	.590	.000	.426	.000	.367	.019
	Max AST	−.001	.000				
	Max urea					−.025	.017
	Max chloride			−.042	.001		
	Max TRIGLYCERIDES			.074	.006		
	Gamma GT					−.002	.019
Demographic	Housewife	−.369	.023				
	No. comorbidities	−.179	.001				
	Insurance Private (yes = 1)					−.962	.020
	Smoking (yes = 1)			.283	.002		
	Live city or town (city = 1)					−.341	.006
	Employment fulltime (yes = 1)			−.213	.025		
Model summary	R Square	.645		.620		.552	
	Adjusted R Square	.630		.604		.534	
	Std Error of the Estimate	.7713		.6964		.8989	

Listed are only the predictors in the final models of the stepwise selection method

Aortic root diameter and basophil levels were significant predictors for the three domains of HRQoL. Patients with large aortic root diameters had lower HRQoL scores, and those with high basophil levels had higher HRQoL scores. Age and LVEF were significant predictors for the physical and social HRQoL scores, where the LVEF was directly proportional to the HRQoL scores and age was inversely proportional to the HRQoL scores. High systolic blood pressure on admission was a significant predictor associated with lower emotional and social scores. Being a housewife, having a large number of comorbidities, presenting with high aspartate aminotransferase (AST) on admission, being older, having high diastolic blood pressure on admission, and presenting with a large septal thickness and aortic root diameter were predictors of lower physical HRQoL scores. Large LVES and LVEF values and high basophil levels were associated with higher physical HRQoL scores. The adjusted coefficients of determination (${\overline{R}}^2$) of the multiple linear regression models were 0.630, 0.604 and 0.534 for the physical, emotional and social HRQoL scores, respectively.

## Discussion

Because one goal of ePCI is to decrease mortality and optimize the HRQoL for CAD patients, it is important to understand how the patients’ clinical and sociodemographic factors before the ePCI, within the context of culture, may affect the HRQoL following the outcome of the procedure. In this study, we evaluated how multiple factors identified for CAD patients at the time of admission correlated with their HRQoL 6 months after their ePCI procedures. To the best of our knowledge, the present study is the first to report HRQoL for Jordanian CAD patients after undergoing ePCI procedures.

Multiple factors were identified that can be used to predict the HRQoL for each domain (physical, emotional and social) 6 months after the ePCI. We developed and validated models based on MLR analyses for predicting the HRQoL of ePCI patients from the data routinely acquired on admission. The results of the developed models show excellent determinations for the three domains of the HRQoL, with adjusted coefficients of determination exceeding 0.5. These models can be used as decision-making tools to guide healthcare professionals as to when it is appropriate to perform ePCIs [22]. Subsequently, the rate of inappropriate ePCIs will decline. Patients who meet certain levels of the determinant factors and for whom the model predicts high HRQoL scores after an ePCI will be recommended to undergo the procedure. Alternatively, patients who do not meet certain levels of the determinant factors and for whom the model predicts poor HRQoL scores after an ePCI will be given two options. The first option is to not undergo the procedure and to stay on medication therapy. This would avoid many inappropriate ePCI operations and reduce costs and adverse effects. The second option, if some factors can be adjusted by medications or lifestyle changes over time, would be to delay the ePCI until the determinant factors reached optimal levels.

### Comparisons to norms

Our finding that females have lower HRQoL scores than males is consistent with other studies [20–23]. Riedinger et al. [24] studied HRQoL in patients with heart failure. They showed that women have lower HRQoL scores than men even after controlling for age and LVEF. A possible explanation for men having higher HRQoL scores than women in our study is that Jordanian men may try to suppress their feelings to show that they can handle the negative consequences ePCIs. This would be consistent with the gender roles in Jordan, specifically, and in the Middle East region in general [25].

LVEF was a significant predictor of the physical and social HRQoL scores after an ePCI, with a positive association. Compared to scores from patients with normal LVEFs, the physical, emotional and social HRQoL scores of patients with reduced LVEF were lower (Fig. 3(a)). The observed lower HRQoL scores with reduced LVEFs indicate that the level of systolic heart function on admission is a determinant for HRQoL 6 months after the ePCI. Similar results were reported by Pettersen et al. for the QoL of acute MI patients 2.5 years after the index date of the MI [26]. Reduced LVEFs may correlate with lower HRQoL scores because patients with lower LVEFs may experience many symptoms, including fatigue, dyspnea and sleep disturbances [27].

Increased septal thickness was significantly correlated with a decrease in both physical and emotional HRQoL scores after the ePCI. This negative association is in accordance with previous studies where septal thickness was used to determine the presence of concentric left ventricle hypertrophy, which causes the heart to fail to pump as much blood as is needed [28].

Surprisingly, patients with stenosis and no stent deployment tended to have similar HRQoL scores as patients without stenosis. It is possible that the medical problem for patients without stenosis who underwent an ePCI was not CAD, so their health-related problems were not solved by the ePCI. Hence, their HRQoL scores after an unnecessary ePCI were low. The physical and emotional HRQoL scores after the ePCI were significantly higher for patients with deployed stents than for those with no stenosis. The patients with stents had their CAD problems identified and solved, resulting in higher HRQoL.

### Determinants of HRQoL

Of the 42 factors studied, we found at least nine significant factors to predict each domain of the HRQoL 6 months after the ePCI. The majority of these factors are from the echocardiography results and laboratory tests, and some of these factors have been previously documented to affect HRQoL in MI patients [7, 26].

One factor shown to affect all HRQoL domains was basophil level, which tended to positively correlate with all domains of HRQoL scores. To the best of our knowledge, we are the first to report this relationship between basophil level and HRQoL after ePCI procedures. Basophils are known inflammatory biomarkers and have been used as an indicator of CAD [29]. A high percentage of basophils on admission indicated the presence of CAD, and an ePCI was performed to treat and relieve the CAD symptoms. Hence, in these patients, the probability of having higher HRQoL after 6 months was high. Patients with a low percentage of basophils might not have had CAD at the time of the ePCI procedure, and the ePCI may have been unnecessary (and unjustified) for those patients. An unnecessary ePCI decreased the probability of having high HRQoL 6 months after the procedure.

Other demographic factors that were also significant determinants for specific domains of HRQoL scores included full-time employment, being a housewife, living in a city, smoking and type of health insurance. Being a fulltime employee tended to decrease the emotional score for patients 6 months after their ePCIs. After undergoing an ePCI, patients may have felt that their bodies were weak, making them less able to tolerate long periods of work and the stress of full-time employment. In contrast, smoking tended to increase the emotional HRQoL score for patients 6 months after their ePCIs. This is most likely because nicotine acts as a moderately effective mood stabilizer and counteracts depression. Patients who smoke can feel temporarily calmer after smoking [30]. However, the dangerous risks of smoking overbalance the benefits.

### Limitations of the study

One limitation of the current study was that the HRQoL was measured only once. Measuring the HRQoL scores of the patients before they undergo the ePCI procedure and comparing them to the scores after they underwent the ePCI procedure can give a more accurate evaluation of the outcomes of the ePCIs. It is possible that some of the HRQoL scores, while low, actually increased following the procedure, indicating that the HRQoL improved. Nevertheless, the encouraging results obtained in this study demonstrated the effectiveness of our models in predicting HRQoL 6 months after an ePCI based on clinical data acquired at admission. One of the factors that increased the strength of the results is that the effect of other serious diseases on the measured HRQoL was eliminated by excluding patients with severe cognitive impairments, cancer, liver failure, kidney failure or chronic obstructive pulmonary disease [25, 31, 32]. Importantly, only patients who decided to undergo an elective PCI to improve their HRQoL were included in the study. Accordingly, it was reasonable to compare the HRQoL of CAD patients after their ePCI procedures. There is a clear need for future research that aims at measuring HRQoL at several time points (e.g., before an ePCI and 6 months and 12 months after the ePCI). Such research is necessary to compare changes in HRQoL and to check the stability and reliability of the models developed in this study for the prediction of long-term HRQoL after ePCIs.

## Conclusions

The aim of this prospective cohort study was to assess the HRQoL of Jordanian CAD patients 6 months after undergoing ePCI procedures. The results showed that the data on multiple clinical and sociodemographic factors collected at admission were significantly correlated with HRQoL 6 months after the ePCIs. Aortic root diameter and basophil levels were significantly correlated with the three domains of HRQoL—physical, emotional and social. Septal thickness, LVEF, systolic blood pressure and age were significantly correlated with two domains of HRQoL. The developed MLR models predicted scores for the three domains of HRQoL 6 months after ePCIs with high accuracy (${\overline{R}}^2$ > 0.5).

## Abbreviations

AST: Aspartate aminotransferase
BMI: Body mass index
BP: Blood pressure
CAD: Coronary artery disease
CI: Confidence interval
ePCI: Elective percutaneous coronary intervention
HRQoL: Health-related quality of life
LOS: Length of stay
LVED: Left ventricle end-diastolic diameter
LVEF: Left ventricle ejection fraction
LVES: Left ventricle end-systolic diameter
MI: Myocardial infarction
PCI: Percutaneous coronary intervention
QLMI-2: Improved MacNew quality of life following acute myocardial infarction
